# A multidimensional construct of helicopter parenting and college students’ game and social media addictive behaviors: A cross-cultural study in South Korea and China

**DOI:** 10.3389/fpsyg.2022.1022914

**Published:** 2023-03-02

**Authors:** Woosang Hwang, Xiaoyu Fu, Seonghee Kim, Eunjoo Jung, Yue Zhang

**Affiliations:** ^1^Department of Human Development and Family Sciences, Texas Tech University, Lubbock, TX, United States; ^2^Department of Human Development and Family Science, Syracuse University, Syracuse, NY, United States; ^3^Research Institute for Liberal Education, Yonsei University, Seoul, Republic of Korea; ^4^Department of Psychology, Santa Clara University, Santa Clara, CA, United States

**Keywords:** helicopter parenting, game addiction, social media addiction, cross-cultural study, China, South Korea, emerging adulthood

## Abstract

**Introduction:**

We explored latent classes of helicopter parenting among Korean and Chinese college students. In addition, we examined whether these latent classes of helicopter parenting are related to Korean and Chinese students’ game and social media addictive behaviors.

**Methods:**

A three-step latent class analysis was conducted using 452 students from six universities in South Korea and 372 students from four universities in China.

**Results and discussion:**

We identified four distinct helicopter parenting latent classes among the parents of Korean and Chinese students: *weak, strong, academic management, and academic and schedule management*. We also found that Korean students in the *strong* class reported significantly higher levels of game and social media addictive behaviors than those in the other three classes, but this did not hold for Chinese students. This finding indicates that the association between helicopter parenting and college students’ game and social media addictive behaviors can be differentiated within Asian cultural contexts.

## Introduction

The prevalence of internet addiction, especially game and social media addictive behaviors, among children has been a concern that was amplified during the COVID-19 pandemic (Lin, 2020; Li et al., 2021). Game addiction is defined as “the excessive and compulsive use of computer or video games that results in social and/or emotional problems; despite these problems, the gamer is unable to control this excessive use” (Lemmens et al., 2009, p. 78). Social media addictive behaviors include the excessive and uncontrollable use of social media, regardless of undesirable consequences in other domains of life (Turel et al., 2018). These addictive behaviors contribute to students’ poor academic performance and well-being (Jiang, 2014; Wang J. et al., 2021). Many studies suggest that parents’ controlling behaviors, including psychological and behavioral control, are associated with increased internet and game addiction (Shek et al., 2018; Lin et al., 2020). Other studies have found that parents’ warmth may serve as a protective factor, reducing the likelihood of college students’ internet addiction (Yao et al., 2014; Zhang et al., 2019). As a parenting style characterized by overinvolvement and overcontrol in children’s life due to care and concern for their well-being, helicopter parenting combines the features of both parental control and parental warmth (Padilla-Walker and Nelson, 2012). Yet, how helicopter parenting affects college students’ game and social media addiction is still unknown.

Parents in Eastern cultures tend to be both caring and controlling with their children and emphasize their academic and career success (Chao and Tseng, 2002). The practice of helicopter parenting is common in many Asian countries, such as China and South Korea (hereafter Korea; Han and Dong, 2009). Studies in the United States have identified multiple dimensions of helicopter parenting, including warmth and controlling behavior, which are associated differently with children’s academic and psychological outcomes (Padilla-Walker and Nelson, 2012; Howard et al., 2022). However, studies of helicopter parenting in Eastern cultures have commonly used a variable-centered approach, so whether different helicopter parenting constructs exist remains unclear. In addition, most studies have focused on a single Eastern culture (Kwon et al., 2016; Lee and Kang, 2018; Leung, 2020; Oh et al., 2021) or, if a comparison is made, it is generally between one Eastern culture and a Western culture (Jung et al., 2020; Su et al., 2022). These approaches fail to capture differences in helicopter parenting between Eastern countries (Bornstein, 2012). Furthermore, no study to date has explored the multidimensional construct of helicopter parenting within and across Eastern cultures. Therefore, using a person-centered approach, in the present study we examined the multidimensional construct of helicopter parenting in Korea and China to determine how different aspects of helicopter parenting impact college students’ addictive behaviors with regard to games and social media.

## Literature review

### Multidimensional characteristics of helicopter parenting

Previous studies have found that helicopter parenting is detrimental to college students’ developmental outcomes (Cui et al., 2022; LeBlanc and Lyons, 2022) and is connected to worse well-being (Schiffrin et al., 2014; Reed et al., 2016; Vigdal and Brønnick, 2022), poor academic outcomes (Jung et al., 2019), increased problem drinking (McGinley and Davis, 2020), and substance use (Cook, 2020). However, other studies have suggested that helicopter parenting can have a positive impact on college students’ life satisfaction and psychological well-being (LeMoyne and Buchanan, 2011; Fingerman et al., 2012) or simply be unrelated to students’ outcomes (Howard et al., 2022). These mixed findings may be explained by the multidimensional nature of helicopter parenting, which consists of autonomy limitation, direct intervention, and the monitoring of children’s academic and daily lives (Luebbe et al., 2018).

Although most studies used helicopter parenting as a general and unidimensional construct (Kwon et al., 2016; Cui et al., 2019; Moilanen and Lynn Manuel, 2019), others have examined the dimensionality of helicopter parenting and found significant differences between the impact of different dimensions (Padilla-Walker et al., 2021; Hwang et al., 2022; Zong and Hawk, 2022). For example, Segrin et al. (2012) identified four subfactors within the helicopter parenting construct, namely, anticipatory problem solving, advice/affect management, child self-direction, and tangible assistance. Continuing this line of thought, Luebbe et al. (2018) found that helicopter parenting is a multidimensional construct, including three aspects of parenting control: information seeking (asking for information about daily life and academic progress), direct intervention (direct involvement in solving interpersonal problems for emerging adult offspring), and limitation of autonomy (structuring emerging adult children’s lives to prevent mistakes). When this is not combined with other controlling behaviors, information-seeking behaviors are perceived by emerging adults as caring and supportive and lead to rational decision-making and better academic functioning. However, both direct intervention and limiting autonomy were related to higher levels of irrational decision-making (Luebbe et al., 2018).

Other studies have explored the multidimensional construct of helicopter parenting in combination with other parenting practices (or perceptions of parenting practices), such as autonomy support (Hwang and Jung, 2021), parental warmth and psychological control (Padilla-Walker et al., 2021), positive parenting (Howard et al., 2022), and emerging adults’ perceptions of overcontrol (Rote et al., 2020). Studies have found that when parental warmth or support co-exists with helicopter parenting behaviors or is perceived by emerging adults, the detrimental effects of helicopter parenting on college students’ outcomes are attenuated (Padilla-Walker et al., 2021; Howard et al., 2022).

However, previous studies exploring the multidimensional construct of helicopter parenting have predominantly been conducted in Western contexts (Rote et al., 2020; Hwang and Jung, 2021; Padilla-Walker et al., 2021). As a result, little is known of the dimensionality of helicopter parenting behaviors in Eastern contexts, where family orientation and parental roles may differ. Families in Eastern contexts emphasize interdependence and relatedness among family members and compliance with parental authority, whereas families in Western contexts emphasize independence and individuality (Chao and Tseng, 2002). Hwang et al. (2022) found that latent class structures of helicopter parenting differed between China and the United States, suggesting a cultural difference in helicopter parenting and its effects on college students’ game and social media addictive behaviors. Therefore, this study explored the similarities and differences in helicopter parenting latent classes in Eastern cultures, which adds to the growing research on helicopter parenting outside of the United States.

### Cultural similarities and differences in helicopter parenting between Korea and China

As both Chinese and Koreans adhere to similar values influenced by Confucianism, such as filial piety and familism (Chao, 2000), their family dynamics and parenting practices are also similar. In Confucian cultures, parents are considered to constitute the authority in a family, and they are responsible for children’s success, even during adulthood (Chao and Tseng, 2002). Strict discipline is considered to come from a place of love and care (Park and Chesla, 2007). Thus, college students in Eastern countries such as Korea and China tend to perceive controlling parenting behaviors as signs of care and support (Chen-Bouck and Patterson, 2017). The coexistence of parental control and care indicates that helicopter parenting is inherent to familial relationships in Eastern cultures (Leung et al., 2018).

Due to the shared cultural values, helicopter parenting behaviors in Korea and China have some similarities. They are characterized by excessive emphasis on academic achievement, in addition to commonly observed helicopter parenting behaviors, such as monitoring children’s social activities, making decisions for them, and providing excessive care (Kwon et al., 2017; Leung and Shek, 2018; Ho et al., 2022). For example, Leung and Shek (2019) found that helicopter parents tended to over-emphasize academic performance in China because it can be considered the only indicator of future success. Similarly, a qualitative study of Korean American college students found that helicopter parents emphasized academic performance due to their high expectations and investment (Kwon et al., 2017). In contrast to the detrimental influence on college students’ psychological and physical well-being typically found in Western cultures, most of these students believed that helicopter parenting could be beneficial to their academic performance and career development (Kwon et al., 2017).

Differences in helicopter parenting behaviors between Korea and China exist because of the social and cultural differences between the two countries. To promote children’s academic achievement and build family pride, Chinese helicopter parents are more likely to compare their children’s performance with that of their peers and overschedule activities for the children (Leung and Shek, 2018). In addition, having only one child might foster helicopter parenting behaviors; parents with one child are more likely to exhibit overprotective and pushy parenting than those with more than one child (Khadaroo and MacCallum, 2021). In China, parents’ investment in and expectations of single children are higher than those for multiple children, which leads to greater pressure to help children succeed (Hu and Shi, 2020). In Korea, however, college students tend to have mixed feelings about helicopter parenting. For example, college students have positive attitudes about parental support during emerging adulthood, whereas they have negative attitudes about parental control for their autonomy (Lee and Kang, 2018). Relatedly, Jung et al. (2019) found that parenting practices were not significantly different between the United States and Korea.

In short, Korean and Chinese parents may have both similarities and differences in their helicopter parenting behaviors due to their cultural commonality. Because previous research has predominantly examined the cultural differences in helicopter parenting between Western and Eastern countries using a dichotomous approach, differences in helicopter parenting between Eastern cultures and their association with children’s outcomes has been neglected. Therefore, the goal of this study is to advance our knowledge of helicopter parenting within and across Eastern countries through an in-depth investigation.

### Differences between Korea and China in helicopter parenting and its relationship to college students’ addictive game and social media use

The prevalence of excessive use of the internet, social media, and games and its detrimental influence on students’ well-being has been well-documented in both Korea and China. More than 80% of adolescents in Korea and China use social media or play computer games, and about 14% of adolescents are addicted (Cui et al., 2018; Wang M. et al., 2021). However, in a cross-cultural study, gaming addiction was found to be more prevalent among Chinese adolescents, at 30.4%, which was more than twice as much as the percentage in Korea (11.4%; Cui et al., 2018). If individuals are addicted, they may be unable to halt their behaviors in spite of the harmful consequences (Hagedorn and Young, 2011). A number of studies suggest that students’ gaming addiction negatively impacts their life through lower self-control (Jeong et al., 2019), insomnia (Lam, 2014), and poor academic performance (Jiang, 2014). Similarly, studies found positive associations between students’ social media addiction and depression (Wang et al., 2018), psychological distress (Wong et al., 2020), poor sleep quality (Wong et al., 2020), and even suicidal behaviors (Sedgwick et al., 2019). The prevalence of students’ addictive behaviors in relation to games and social media, along with their unfavorable effects on students’ outcomes, indicate the need for further investigation of the potential causes of this phenomenon.

The role helicopter parenting plays in students’ addiction to games and social media can be understood in light of family systems theory. The family environment is the primary context of children’s growth and development, and it generally plays a significant role in students’ addictive behaviors. Family systems theory views the family as a whole as an overarching system with various subsystems consisting of interdependent relationships between family members (Cox and Paley, 1997). This theory suggests that to obtain a complete understanding of individual behavior, the roles of family subsystems and their interrelations, such as parent–child relationships, should be considered (Cox and Paley, 1997). Family subsystems function differently across families when they respond to children’s developmental needs, and they yield different consequences (Lander et al., 2013). Studies indicate that certain parenting practices, including parental warmth, attachment, and responsiveness, are associated with fewer internet addictive behaviors (Kim and Kim, 2015; Zhang et al., 2019), whereas a dysfunctional parent–child relationship leads students to extensive internet use to escape (Shek et al., 2018; Sun and Wilkinson, 2020; Zhao et al., 2022). Parental behavioral and psychological control were found to be robust indicators of internet gaming disorder and internet addiction among Chinse adolescents in two longitudinal studies (Shek et al., 2018; Lin et al., 2020). Since helicopter parenting possesses the attributes of parental warmth and control (Padilla-Walker and Nelson, 2012), it is unclear how it impacts college students’ game and social media addictive behaviors. Studies have shown that strong helicopter parenting is associated with low self-control in college students, which may result in a failure to resist the temptation to play games and use social media uncontrollably (Li et al., 2014; Hong and Cui, 2020; Love et al., 2020). Although few studies have examined the impact of helicopter parenting on students’ addictive behaviors with respect to games and social media, parents’ overinvolvement and overprotective behaviors have been found to be closely tied to problematic gaming and internet addictive behaviors in both Korea (Jeong et al., 2019) and China (Zhang et al., 2019). In a cross-cultural study, the lack of parental attachment and mediation were found to have a stronger influence on problematic gaming for adolescents in China than those in Korea, which indicates that cultural differences may exist in the association between parenting practice and addictive behaviors (Cui et al., 2018).

Family systems theory emphasizes that relationships between mother, father, and child are interdependent (Minuchin, 1985). One parent could therefore affect the relationship between the other parent and the child (Caldera and Lindsey, 2006; Stroud et al., 2011), and the parenting style of one parent could affect the other’s style (Winsler et al., 2005; Tavassolie et al., 2016). However, previous helicopter parenting studies using a person-centered approach did not address how multidimensional constructs, including both maternal and paternal helicopter parenting, are associated with college students’ game and social media addictive behaviors. Therefore, this study focused on the above associations in Korea and China.

### Aims and hypotheses

This study’s first aim was to uncover helicopter parenting latent classes among Korean and Chinese college students. We hypothesized that distinct helicopter parenting latent classes (including strong and weak latent classes) would be identified among Korean and Chinese college students. The second aim was to examine whether helicopter parenting latent classes would be related to Korean and Chinese college students’ game and social media addictive behaviors. We hypothesized that Korean and Chinese college students in the strong helicopter parenting classes would report higher levels of addictive behaviors in relation to games and social media than those in other helicopter parenting classes.

## Method

### Participants

Data for this study were sampled from a larger helicopter parenting survey (Hwang et al., 2022). After receiving Institutional Review Board approval, we contacted college students studying at six urban universities in Korea and four urban universities in China. We recruited college students through their course instructors in humanities and social science classes from April 2019 to October 2020. We translated the Korean version of the survey instrument into Chinese using the back-translation method (van De Vijver and Leung, 1997). In China, we provided an online survey link to college students *via* course instructors. In Korea, we provided college students either an online survey link or a paper questionnaire based on the course instructors’ preference. The college students who completed both the consent form and the survey received extra credit on their semester grades as compensation for their participation in the study.

In total, 685 Korean and 709 Chinese college students participated in the study. The response rates were 73% in Korea (*n* = 500) and 78% in China (*n* = 553). As young adulthood is considered to be between 18 and 25 years of age (Twenge et al., 2019), we selected from these responses those of 452 Korean college students and 372 Chinese college students (1) whose ages ranged between 18 and 25 years, (2) who had two surviving parents (both mother and father), and (3) who responded to both maternal and paternal helicopter parenting measures.

### Measures

#### Helicopter parenting

Maternal and paternal helicopter parenting were measured using nine items of the Helicopter Parenting Behaviors scale (Schiffrin et al., 2014). Example items were “my mother/father had/will have a say in what major I chose/will choose” and “my mother/father calls me to track my schoolwork.” Responses were given on a Likert-type scale ranging from (1) *strongly disagree* to (6) *strongly agree*. Cronbach’s alpha values were computed and ranged from 0.81 to 0.84 in maternal and paternal helicopter parenting in Korean and Chinese college students. We dichotomized all maternal and paternal helicopter parenting items into low (from 1 = *strongly disagree* to 3 = *somewhat disagree*) and high (from 4 = *somewhat agree* to 6 = *strongly agree*) categories and used them as indicators of parents’ helicopter parenting in the latent class analysis. Dichotomization was performed because scores on a large number of helicopter parenting items were skewed in both the Korean and Chinese samples (the skewed items are presented in Table 1). These skewed items would not completely capture the full range of the scale. Dichotomization was performed because categorical indicators create better class separation and interpretation than skewed ordinal indicators (DeCoster et al., 2009; Macia and Wickham, 2019; Vasilenko, 2021; Hwang and Jung, 2022).

**Table 1:** Descriptive results of maternal and paternal helicopter parenting in Korean and Chinese college students.

Helicopter parenting items (range: 1–6)	Korean college students (*n* = 452)				Chinese college students (*n* = 372)			
	Mother		Father		Mother		Father	
	*n* (*%*)	*M* (*SD*)	*n* (*%*)	*M* (*SD*)	*n* (*%*)	*M* (*SD*)	*n* (*%*)	*M* (*SD*)
Mean score of nine items		2.61 (0.91)		2.30 (0.91)		2.82 (0.94)		2.64 (0.89)
1. Advise my college major		3.75 (1.51)		3.47 (1.56)		3.42 (1.45)		3.43 (1.43)
Low category	174 (38.5)		211 (46.7)		169 (45.4)		173 (46.5)	
High category	278 (61.5)		241 (53.3)		202 (54.3)		198 (53.2)	
2. Monitor my exercise schedule		2.35 (1.39)^a^		2.09 (1.28)^b^		2.79 (1.42)		2.69 (1.40)
Low category	346 (76.5)		382 (84.5)		238 (64.0)		252 (67.7)	
High category	106 (23.5)		70 (15.5)		132 (35.5)		118 (31.7)	
3. Call parents to know where I am		3.66 (1.57)		2.95 (1.61)		2.87 (1.57)		2.54 (1.44)^a^
Low category	191 (42.3)		280 (61.9)		232 (62.4)		269 (72.3)	
High category	261 (57.7)		172 (38.1)		139 (37.4)		100 (26.9)	
4. Monitor my diet		2.31 (1.38)^a^		1.93 (1.20)^b^		3.24 (1.53)		2.77 (1.47)
Low category	354 (78.3)		394 (87.2)		191 (51.3)		247 (66.4)	
High category	98 (21.7)		58 (12.8)		179 (48.1)		123 (33.1)	
5. Monitor who I spent time with		2.62 (1.43)		2.14 (1.31)^b^		2.91 (1.50)		2.62 (1.39)^a^
Low category	318 (70.4)		377 (83.4)		237 (63.7)		266 (71.5)	
High category	133 (29.4)		74 (16.4)		133 (35.8)		102 (27.4)	
6. Call me to track my schoolwork		2.17 (1.37)^a^		1.91 (1.26)^b^		2.82 (1.64)		2.61 (1.51)^a^
Low category	363 (80.3)		390 (86.3)		230 (61.8)		258 (69.4)	
High category	89 (19.7)		62 (13.7)		139 (37.4)		111 (29.8)	
7. Call professor about a low grade		1.65 (1.16)^b^		1.60 (1.13)^b^		1.91 (1.18)^b^		1.87 (1.13)^b^
Low category	412 (91.2)		411 (90.9)		325 (87.4)		329 (88.4)	
High category	40 (8.8)		41 (9.1)		44 (11.8)		39 (10.5)	
8. Intervene my roommate issue		1.98 (1.26)^b^		1.76 (1.13)^b^		1.81 (1.08)^b^		1.71 (0.96)^b^
Low category	385 (85.2)		401 (88.7)		336 (90.3)		347 (93.3)	
High category	66 (14.6)		50 (11.1)		34 (9.1)		21 (5.6)	
9. Have a curfew every night		3.01 (1.71)		2.93 (1.75)		3.64 (1.83)		3.58 (1.78)
Low category	254 (56.2)		274 (60.6)		155 (41.7)		161 (43.3)	
High category	197 (43.6)		177 (39.2)		215 (57.8)		207 (55.6)	

a Moderately skewed.

b Strongly skewed.

#### Game and social media addictive behaviors

Addictive gaming behaviors were measured with the seven items of the Game Addiction Scale (Lemmens et al., 2009). Example items were “I have played games to forget about real life” and “Others have unsuccessfully tried to reduce my time spent on games.” Responses were given on a Likert-type scale ranging from (1) *strongly disagree* to (5) *strongly agree*. The Cronbach’s alpha value was 0.90 in Korean college students and 0.81 in Chinese college students. We used the mean score of the seven items in the analysis.

Social media addictive behaviors were measured with nine items using the Social Networking Site Addiction Symptoms Scale (Osatuyi and Turel, 2018). Example items were “I think that I was addicted to the social networking website” and “I often failed to get enough rest because I interacted with the social networking website.” Responses were given on a Likert-type scale ranging from (1) *strongly disagree* to (5) *strongly agree*. The Cronbach’s alpha value was 0.92 in Korean college students and 0.87 in Chinese college students. We used the mean score of the nine items in the analysis.

#### Control variables

Previous studies have found that age and gender are closely related to gaming and social media addictive behaviors (Andreassen et al., 2016; Su et al., 2020). For example, age is inversely related to gaming and social media addictive behaviors (Andreassen et al., 2016). Regarding gender, men are more likely to display behavior indicating addiction to computer games behaviors than women, whereas women are more likely to manifest behaviors reflecting social media addiction than men (Su et al., 2020). Based on these considerations, we used college students’ ages and genders (0 = *male*, 1 = *female*) as control variables.

### Analytic strategy

We used Latent Gold 6.0 (Vermunt and Magidson, 2021) to conduct a latent class analysis using 18 dichotomized maternal and paternal helicopter parenting indicators in Korea and China. Latent class analysis allows the identification of unobserved subgroups within Korean and Chinese college students based on their responses to maternal and paternal helicopter parenting indicators (Nylund-Gibson and Choi, 2018). Based on this analysis, we identified helicopter parenting latent classes in Korean and Chinese college students. We used the Bayesian Information Criterion (BIC), the Consistent Akaike Information Criterion (CAIC), Entropy, and the Vuong and Lo–Mendell–Rubin Likelihood-Ratio Test (VLMR-RLT). In this procedure, the latent class model with the smallest BIC, AIC, and SABIC values and entropy values over 0.8 is the best-fitting solution (Nylund-Gibson and Choi, 2018). The VLMR-RLT assesses whether adding a class leads to a significant improvement in model fit, and a non-significant VLMR-RLT value for a k-class model explains that the k − 1 class model is the best-fitting model (Nylund-Gibson and Choi, 2018).

When the optimal number of latent classes was identified, we employed a three-step Bolck-Croon Hagenaars approach (Bolck et al., 2004) to determine what classes were associated with game and social media addictive behaviors. This method is beneficial for adjusting classification errors to reduce bias (Bakk and Vermunt, 2015). In this stage, regression analyses of the association between class membership, game and social media addictive behaviors, and the control variables (age and gender) were conducted, with analyses weighted by latent class membership probability. Regression analyses for game-related addictive behaviors (Model 1 in Korean college students and Model 3 in Chinese college students) and social media addictive behaviors (Model 2 in Korean college students and Model 4 in Chinese college students) were separately conducted. To address the missing values, we used full-information maximum likelihood estimation (Vermunt and Magidson, 2021).

## Results

### Descriptive analyses

Descriptive statistics for the demographic characteristics and outcome variables are presented in Table 2. The mean ages of the Korean and Chinese college students were 19 and 20 years old (ranging from 18 to 25 years), respectively, and the majority had living biological parents (97.8% of Korean college students and 98.9% of Chinese college students). Most of their parents were married (88.3% of Korean and 93.5% of Chinese college students). Descriptive statistics regarding maternal and paternal helicopter parenting in Korean and Chinese college students are presented in Table 1. The results showed that the mean score of nine maternal helicopter parenting items was higher than the mean score of nine paternal helicopter parenting items in both Korean college students (*M* = 2.63 in maternal helicopter parenting; *M* = 2.30 in paternal helicopter parenting) and Chinese college students *M* = 2.82 in maternal helicopter parenting; *M* = 2.64 in paternal helicopter parenting.

**Table 2:** Descript results of demographic characteristics and game and social media addictive behaviors in Korean and Chinese college students.

Variables		Korean college students (*n* = 452)		Chinese college students (*n* = 372)	
	Range	*n* (*%*)	*M* (*SD*)	*n* (*%*)	*M* (*SD*)
**Demographic characteristics**					
Age	18–25		20.78 (1.88)		19.63 (1.48)
**Gender**					
Male		159 (35.2)		63 (16.9)	
Female		292 (64.6)		305 (82.0)	
**Having a sibling**					
Yes		410 (90.7)		219 (58.9)	
No		41 (9.1)		146 (39.2)	
**Current living arrangement**					
Living with parents		275 (60.8)		2 (0.5)	
Living separately from parents		176 (38.9)		370 (99.5)	
**Mother–child relations**					
Biological mother		446 (98.7)		366 (98.4)	
Stepmother		4 (0.9)		6 (1.6)	
**Father–child relations**					
Biological father		442 (97.8)		368 (98.9)	
Stepfather		8 (1.8)		4 (1.1)	
Mother’s education	1–4		2.22 (1.01)		1.51 (0.90)
Father’s education	1–4		2.42 (1.01)		1.64 (1.00)
**Parents’ marital status**					
Married		399 (88.3)		348 (93.5)	
Others		46 (10.2)		22 (5.9)	
**Outcome variables**					
Game addictive behaviors	1–5		1.62 (0.82)		2.08 (0.78)
Social media addictive behaviors	1–5		2.13 (0.91)		2.19 (0.77)

### Helicopter parenting classes

Helicopter parenting items were originally measured with ordinal response categories. We initially treated these 18 items (9 for maternal helicopter parenting and 9 for paternal helicopter parenting) as continuous and conducted a latent profile analysis to define classes within them. However, the latent classes were not well-differentiated in the best-fitting solution. Therefore, we concluded that latent class analysis with dichotomous indicators was the best method for analyzing the data.

**Figure 1:**
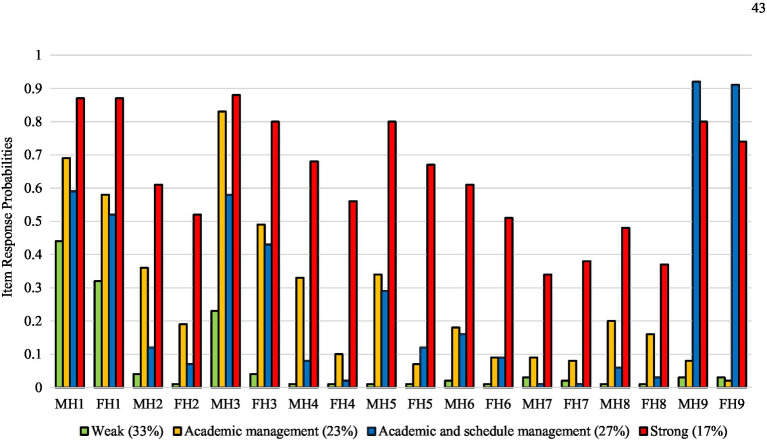
Item response and latent class probabilities of four religiosity classes in Korean college students. MH, Maternal helicopter parenting item; FH, Paternal helicopter parenting item. Item 1, Advise my college major; Item 2, Monitor my exercise schedule; Item 3, Call parents to know where I am; Item 4, Monitor my diet; Item 5, Monitor who I spent time with; Item 6, Call me to track my schoolwork; Item 7, Call professor about a low grade; Item 8, Intervene my roommate issue; Item 9, Have a curfew every night.

**Figure 2:**
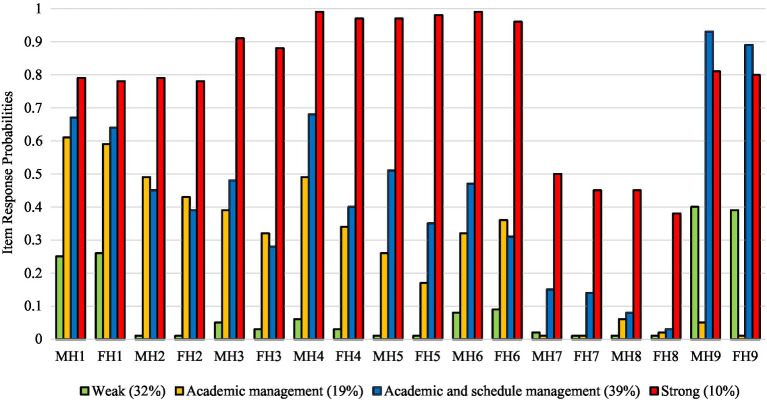
Item response and latent class probabilities of four religiosity classes in Chinese college students. MH, Maternal helicopter parenting item; FH, Paternal helicopter parenting item. Item 1, Advise my college major; Item 2, Monitor my exercise schedule; Item 3, Call parents to know where I am; Item 4, Monitor my diet; Item 5, Monitor who I spent time with; Item 6, Call me to track my schoolwork; Item 7, Call professor about a low grade; Item 8, Intervene my roommate issue; Item 9, Have a curfew every night.

The results of latent class analyses for Korean and Chinese college students are presented in Table 3. The smallest BIC and CAIC values, non-significant VLMR-RLT values, and entropy values over 0.8 indicated that a four-class model had the best fit for both Korean and Chinese college students. The item response and latent class probabilities for these four helicopter parenting classes are presented in Figure 1 (Korean college students) and Figure 2 (Chinese college students). Using 0.5 as a cutoff to define classes from item response probabilities, we labeled the four classes based on a previous study (Hwang et al., 2022). The first latent class was *weak*. In this class, item response probabilities in all indicators were under 0.5 in Korean and Chinese college students. Regarding latent class probabilities, that for Korean college students was 33%, and for Chinese college students it was 32%. The second latent class was *academic management*. In this class, item response probabilities in mothers’ and fathers’ “advise my college major” indicators were over 0.5 in Korean and Chinese college students. Regarding latent class probabilities, Korean college students’ were 23%, and Chinese college students’ were 19%. The third class was *academic and schedule management*. In this class, item response probabilities in mothers’ and fathers’ “advise my college major” and “have a curfew every night” indicators were over 0.5 in Korean and Chinese college students. Regarding latent class probabilities, Korean college students’ were 27%, and Chinese college students’ were 39%. The fourth latent class was *strong*. In this class, item response probabilities in all indicators were over 0.5 except for mothers’ and fathers’ “call professor about a low grade” and “intervene regarding my roommate issue” indicators in Korean and Chinese college students. Regarding latent class probabilities, Korean college students’ were 17%, and Chinese college students’ were 10%.

**Table 3:** Latent class analysis statistics and fit indices.

	Korean college students (*n* = 452)				Chinese college students (*n* = 372)			
Class (*n*)	BIC	CAIC	VLMR	Entropy	BIC	CAIC	VLMR	Entropy
1	8471.22	8489.22	-	-	7753.06	7771.06	-	-
2	7557.59	7594.59	*p* < 0.001	0.84	6963.30	7000.30	*p* < 0.001	0.85
3	7425.87	7481.87	*p* < 0.001	0.86	6794.66	6850.66	*p* < 0.001	0.86
4	**7332.40**	**7407.40**	*p* < 0.001	0.85	6742.74	**6817.74**	*p* < 0.001	0.88
5	7335.96	7429.96	*p* > 0.05	0.88	**6728.71**	6822.71	*p* > 0.05	0.88
6	7354.77	7467.77	*p* < 0.05	0.88	6733.09	6846.09	*p* < 0.001	0.90

Bolded values indicate best fit for each respective statistic. BIC, Bayesian Information Criterion; CAIC, Consistent Akaike Information Criterion; VLMR, Vuong-Lo–Mendell–Rubin Test.

Given that the same four latent classes were identified for Korean and Chinese college students, we conducted a chi-square test to determine whether the distributions of the four latent classes differed between Korean and Chinese college students. Results showed that the latent class probabilities did not significantly differ between the two groups. We conclude that the measurement and representation of helicopter parenting were equivalent between the two groups.

### Associations between helicopter parenting latent classes and game and social media addictive behaviors

The results of regression analyses are presented in Table 4. In Models 1 and 2, Korean college students in the weak, academic management, and academic and schedule management classes reported significantly lower levels of addictive behaviors concerning games and social media than those in the *strong* latent class. These results indicate that Korean college students who perceived strong helicopter parenting from their mothers and fathers were more likely to exhibit addictive behaviors concerning games and social media than those who perceived the other three types of helicopter parenting from their mothers and fathers. Contrary to our expectations, however, the above associations were not found in Chinese college students. In Models 3 and 4, Chinese college students’ addictive behaviors concerning games and social media were not significantly different across the four helicopter parenting classes.

**Table 4:** Associations of helicopter parenting latent class membership with game and social media addictive behaviors.

	Korean college students (*n* = 452)			
	Model 1 Game addictive behaviors		Model 2 Social media addictive behaviors	
Variables	*b* (*se*)	z	*b* (*se*)	z
Intercept	1.91 (0.43)	4.45^***^	3.04 (0.49)	6.10^***^
**Class memberships (reference: strong)**				
Weak	−0.50 (0.12)	−3.96^***^	−0.69 (0.13)	−5.06^***^
Academic management	−0.31 (0.13)	−2.34^*^	−0.68 (0.14)	−4.64^***^
Academic and schedule management	−0.28 (0.14)	−1.89^†^	−0.36 (0.15)	−2.37^*^
**Control variables**				
Age	0.01 (0.01)	0.89	−0.03 (0.02)	−1.44
Female (vs. male)	−0.52 (0.08)	−5.85^***^	0.40 (0.09)	4.21^***^
	Chinese college students (*n* = 372)			
	Model 3 Game addictive behaviors		Model 4 Social media addictive behaviors	
	*b* (*se*)	z	*b* (*se*)	z
Intercept	2.24 (0.53)	3.86^***^	2.19 (0.61)	3.58^***^
**Class memberships (reference: strong)**				
Weak	0.05 (0.14)	−0.80	−0.01 (0.14)	−0.08
Academic management	−0.10 (0.15)	−0.17	−0.08 (0.14)	0.58
Academic and schedule management	−0.02 (0.16)	0.27	0.01 (0.16)	−0.08
**Control variables**				
Age	0.01 (0.02)	0.21	−0.00 (0.03)	−0.06
Female (vs. male)	−0.40 (0.11)	−3.49^***^	0.08 (0.11)	0.76

† *p* = 0.058;

* *p* < 0.05;

** *p* < 0.01;

*** *p* < 0.001.

We conducted paired comparisons to investigate other contrasts in the helicopter parenting classes that were not captured by the regression analysis (see Table 5). In the Korean college students, we found that the predicted mean score for social media addictive behaviors in the *academic and schedule management* classes was significantly higher than in the *weak* and *academic management* classes. In the Chinese students, we found no significant differences between the four helicopter parenting latent classes not shown in the regression results.

**Table 5:** Results of paired comparison test of game and social media addictive behaviors across four helicopter parenting latent classes in Korean and Chinese college students.

Outcome variables	Class 1 Strong	Class 2 Weak	Class 3 Academic management	Class 4 Academic and schedule management	*p*-value
	Predicted mean (*SE*)	Predicted mean (*SE*)	Predicted mean (*SE*)	Predicted mean (*SE*)	
**Korean college students**					
Game addictive behaviors (Model 1)	1.94 (0.11)	1.43 (0.06)	1.62 (0.07)	1.65 (0.09)	1 > 2^***^; 1 > 3^*^; 1 > 4^a^; 3 > 2^*^; 4 > 2^b^
Social media addictive behaviors (Model 2)	2.63 (0.11)	1.94 (0.07)	1.95 (0.08)	2.27 (0.09)	1 > 2, 3^***^; 1 > 4^*^; 4 > 2^**^; 4 > 3^*^
**Chinese college students**					
Game addictive behaviors (Model 3)	2.10 (0.12)	2.16 (0.06)	1.99 (0.07)	2.08 (0.09)	-
Social media addictive behaviors (Model 4)	2.22 (0.12)	2.21 (0.06)	2.14 (0.07)	2.24 (0.10)	-

Control variables are not presented.

a *p* = 0.058;

b *p* = 0.057;

* *p* < 0.05;

** *p* < 0.01;

*** *p* < 0.001.

## Discussion

Over the past decade, many researchers have focused on the effects of helicopter parenting on college students’ psychological well-being in various cultural contexts (Cui et al., 2019; Jung et al., 2019). However, less is known about whether helicopter parenting is related to college students’ addictive behaviors in relation to games and social media. Furthermore, although several cross-cultural studies investigated differences in the association between helicopter parenting and college students’ psychological well-being between the United States and Asian countries (Jung et al., 2020; Su et al., 2022), to the best of our knowledge, no study has compared the association between helicopter parenting and college students’ addictive behaviors in relation to games and social media within Asian countries. Using a latent class analysis, we investigated latent classes of helicopter parenting among Korean and Chinese college students to determine whether latent classes of helicopter parenting were associated with Korean and Chinese college students’ addictive behaviors concerning games and social media and how these associations differed between the two countries.

The first hypothesis that distinct helicopter parenting latent classes would be identified among Korean and Chinese college students was supported. We found two consistent polar classes (*weak* and *strong*) and two intermediate classes (*academic management* and *academic and schedule management*) in both Korean and Chinese college students. The *academic management* and *academic and schedule management* classes were distinct from the *weak* and *strong* classes, in which mothers and fathers were mainly involved in their young adult children’s academic issues and personal schedules. These findings are consistent with our expectation that helicopter parenting can manifest in both uniform and non-uniform ways across multiple dimensions (Luebbe et al., 2018; Padilla-Walker et al., 2021; Howard et al., 2022; Hwang and Jung, 2022). In addition, considering that Asian societies tend to emphasize academic success more than Western societies (Hwang and Kim, 2016), two intermediate classes adequately represent the characteristics of an educational achievement-oriented parenting style in the relationship between parents and young adult children in Korea and China.

However, we could not find any heterogeneous combinations of helicopter parenting, such as strong maternal helicopter parenting but weak paternal helicopter parenting classes. This finding indicates that mothers and fathers are likely to show a similar pattern of helicopter parenting. It appears that Korean and Chinese mothers and fathers likely have consensus about helicopter parenting. Interestingly, our results showed that the item response probabilities of maternal helicopter parenting were somewhat higher than paternal helicopter parenting in four latent classes in both Korean and Chinese college students. This finding is consistent with previous studies indicating that mothers are more involved in children’s lives than fathers (Finley et al., 2008; Mailhot and Feeney, 2017).

Consistent with the second hypothesis, we found that Korean college students in the *strong* class reported significantly higher levels of game and social media addictive behaviors than those in the other classes. This finding indicates that strong levels of helicopter parenting could be a risk factor for increasing game and social media addictions. This finding echoes previous findings that excessive parental control is closely related to college students’ risky behaviors (Cook, 2020; McGinley and Davis, 2020). Furthermore, we found that Korean college students in the *academic and schedule management* class reported significantly higher levels of gaming and social media addictive behaviors than those in the *weak* and *academic management* classes. Given that more Korean college students in the *academic and schedule management* class reported a strict curfew by their parents than those in *weak* and *academic management* classes, it is possible that parental schedule control could be related to college students’ social media addictive behaviors in Korea. For example, curfew is considered one of the main predictors of parent–child conflict in Korea (Park, 2016). Therefore, it is possible that a strict curfew leads college students to feel stress regarding parental control. Related, Jeong et al. (2019) found that excessive parental control is significantly associated with college students’ problematic gaming and internet addictive behaviors. Our findings suggest that gaming and social media addictive behaviors could be differently influenced by different helicopter parenting latent classes.

Contrary to our expectations, however, Chinese college students’ gaming and social media addictive behaviors were not significantly different across four classes of helicopter parenting. The result could be linked to Su et al.’s (2022) argument that helicopter parenting is common in China, due to the child-centered family norms under the previous one-child policy. In other words, helicopter parenting would be considered an acceptable parenting style for Chinese college students. Therefore, we speculate that Chinese college students may not perceive helicopter parenting to be intensive parental control. However, researchers have found that, unlike Chinese college students, Korean young adults are ambivalent about helicopter parenting (Kwon et al., 2017; Lee and Kang, 2018). This may be related to the fact that Korean college students are exposed in mixed cultural contexts: a coexistence between personalism based on Westernized culture and familism based on traditional social norms (Kim, 2022). Relatedly, Zhang et al. (2005) found that Korean college students had a lower level of Confucian values than Chinese college students, which led to a cultural difference between Korean and Chinese college students. Thus, it is not surprising that the associations between helicopter parenting latent classes and college students’ game and social media addictive behaviors differed between the two Asian cultural contexts examined in our study.

### Limitations

This study has several limitations. First, our analysis relied on college students’ self-reports of their mothers’ and fathers’ helicopter parenting. Thus, we considered only college students’ perceptions of maternal and paternal helicopter parenting, which may be biased relative to parents’ perceptions of their own and their spouses’ helicopter parenting. In addition, female college students were overrepresented because participant recruitment was conducted in humanities and social science classes. As a result, college students majoring in the areas of science, technology, engineering, and mathematics were underrepresented. For this reason, we controlled for college students’ gender in the analysis, which prevented us from explaining the differences between helicopter parenting and addictive behaviors related to games and social media as a function of parent–child gender combinations. Second, this study was cross-sectional, so we could not investigate the causal association between types of helicopter parenting and college students’ game and social media addictive behaviors.

### Implication

This study has salient practical implications. First, for college counselors and clinicians, the findings indicate the need for an appropriate cultural approach in Asian contexts. Asian countries each have their own specific features of parenting behaviors. This might lead to significant differences in the acceptability of helicopter parenting between the two cultures. Thus, college counselors and clinicians should be aware of country-specific characteristics when dealing with college students. Second, parental education and interventions regarding the appropriate boundaries between independence and dependence in emerging adulthood should be provided, and awareness of the adverse effects of helicopter parenting on game and social media addiction for Korean students and parents should be increased. In this way, those who work with Korean parents and college students could help students to flourish during emerging adulthood. In addition, institutions training counselors in parental or addiction interventions might emphasize instruction regarding the role of helicopter parenting in different Asian countries. Policymakers could consider the need to expand the awareness of game and social media addiction and to implement parent education on emerging adult children.

### Conclusion

This study identified associations between patterns of helicopter parenting and gaming and social media addictive behaviors in Asian contexts. In particular, it indicates the role helicopter parenting behaviors play in addiction issues in Korean college students, but did not find differences among helicopter parenting classes in the Chinese sample. This raises the need to consider cultural differences between Asian countries and simultaneously calls for longitudinal research. The results of this study imply that there might be different perceptions of helicopter parenting by college-aged adults in different Asian countries.

## Data availability statement

The raw data supporting the conclusions of this article will be made available by the authors, without undue reservation.

## Ethics statement

The studies involving human participants were reviewed and approved by Institutional Review Board, Syracuse University Institutional Review Board, Yonsei University. The patients/participants provided their written informed consent to participate in this study.

## Author contributions

WH designed and executed the study, managed data collection, performed the data analysis, and wrote the manuscript. XF, SK, and EJ collaborated with data collection and writing of the study. YZ assisted with data collection. All authors contributed to the article and approved the submitted version.

## Funding

This work was supported by the Ministry of Education of the Republic of Korea and the National Research Foundation of Korea (NRF-2022S1A5C2A04093488).

## Conflict of interest

The authors declare that the research was conducted in the absence of any commercial or financial relationships that could be construed as a potential conflict of interest.

## Publisher’s note

All claims expressed in this article are solely those of the authors and do not necessarily represent those of their affiliated organizations, or those of the publisher, the editors and the reviewers. Any product that may be evaluated in this article, or claim that may be made by its manufacturer, is not guaranteed or endorsed by the publisher.
